# Reirradiation for Nasal Cavity or Paranasal Sinus Tumor—A Multi-Institutional Study

**DOI:** 10.3390/cancers13246315

**Published:** 2021-12-16

**Authors:** Hideya Yamazaki, Gen Suzuki, Norihiro Aibe, Makoto Yasuda, Hiroya Shiomi, Ryoong-Jin Oh, Ken Yoshida, Satoaki Nakamura, Koji Konishi, Mikio Ogita

**Affiliations:** 1 Department of Radiology, Graduate School of Medical Science, Kyoto Prefectural University of Medicine, 465 Kajiicho Kawaramachi Hirokoji, Kamigyo-ku, Kyoto 602-8566, Japan; gensuzu@koto.kpu-m.ac.jp (G.S.); a-ib-n24@koto.kpu-m.ac.jp (N.A.); 2 Department of Otolaryngology-Head and Neck Surgery, Graduate School of Medical Science, Kyoto Prefectural University of Medicine, 465 Kajiicho Kawaramachi Hirokoji, Kamigyo-ku, Kyoto 602-8566, Japan; myasu@koto.kpu-m.ac.jp; 3 CyberKnife Center, Soseikai General Hospital, Kyoto 612-8473, Japan; shiomi@radonc.med.osaka-u.ac.jp; 4 Department of Radiation Oncology, Miyakojima IGRT Clinic, Osaka 534-0021, Japan; oh@osaka-igrt.or.jp; 5 Department of Radiology, Kansai Medical University, Hirakata 573-1191, Japan; yoshidkn@takii.kmu.ac.jp (K.Y.); satoaki@nakamura.pro (S.N.); 6 Department of Radiation Oncology, Osaka International Cancer Institute, Osaka 541-8567, Japan; konisi-ko@mc.pref.osaka.jp; 7 Radiotherapy Department, Fujimoto Hayasuzu Hospital, Miyakonojo 885-0055, Japan; ogita@fujimoto.or.jp

**Keywords:** nasal cavity and paranasal sinus, reirradiation, optic pathway

## Abstract

**Simple Summary:**

Nasal cavity or paranasal sinus tumors are rare, with treatment outcomes reported as a small subgroup of all head and neck tumors. However, due to their unique anatomical characteristics (proximity to the optic pathway and skull base), treatment efficacy and toxicity in should be focused on those areas. After curative treatment involving radiotherapy, local recurrence is common and treated mainly through surgery. Unfortunately, surgery is difficult in almost all cases due to the critical organs involved. Systemic chemotherapy plays a central role in managing recurrent tumors; however, its efficacy is insufficient to extend longevity. Reirradiation is challenging, as it leads to higher toxicity, but it potentially offers long-lasting tumor control, including cure and symptom palliation in patients with no other remaining therapeutic options. We examined this subgroup using multi-institutional data and found that reirradiation is feasible for treating nasal cavity or paranasal sinus tumors. A relatively high distant metastasis ratio was found after reirradiation, especially in patients with lymph node metastasis.

**Abstract:**

We evaluated the efficacy and toxicity of reirradiation of nasal cavity or paranasal sinus tumors. We collected and analyzed multi-institutional data of reirradiation cases. Seventy-eight patients with nasal or paranasal sinus tumors underwent reirradiation. The median survival time was 20 months with a medial follow-up of 10.7 months. The 2-year local control and overall survival rates were 43% and 44%, respectively. Tumor volume (≤25 cm^3^), duration between previous radiotherapy and reirradiation (≤12 months), histology (squamous cell carcinoma), male sex, and lymph node involvement were predisposing factors for poor survival. Distant metastasis was observed in 20 patients (25.6%). Grade ≥ 3 adverse events were observed in 22% of the patients, including five grade 4 (8.6%) cases and one grade 5 (1.2%) case. Tumor location adjacent to the optic pathway was a significant predisposing factor for grade ≥3 visual toxicity. Reirradiation of nasal and paranasal sinus tumors is feasible and effective. However, adverse events, including disease-related toxicities, were significant. Prognostic factors emerge from this study to guide multidisciplinary approaches and clinical trial designs.

## 1. Introduction

Nasal cavity or paranasal sinus malignancies are rare, accounting for 3–5% of head and neck malignancies [1]. Squamous cell carcinoma (SCC) is the most common histological type, followed by adenocarcinoma, olfactory neuroblastoma, adenoid cystic carcinoma, melanoma, and undifferentiated carcinoma [2]. As distant metastasis is generally rare, excluding adenoid cystic carcinoma and olfactory neuroblastoma cases, local recurrence is the main cause of morbidity [3]. Although salvage surgical resection is the preferred treatment combined with chemotherapy with or without radiotherapy, it is occasionally difficult owing to extensive tumor spread to critical organs. Chemotherapy plays a central role in unresectable cases; however, it provides only a non-durable response rate of 10–40% and results in short longevity of less than one year (median survival time: 5–9 months) [4]. Reirradiation could be a viable option to improve survival time and palliation, but it remains challenging because of the associated severe adverse reactions [5,6]. Several trials of reirradiation with or without chemotherapy have been attempted [7,8]; however, they treated head and neck lesions in a heterogeneous population including oral, pharyngeal, laryngeal cancer and more. Therefore, owing to the rare nature of this disease, literature regarding reirradiation of the nasal or paranasal cavity is limited [9,10]. These tumors have a unique characteristic of spreading to vital structures, such as the skull base or the optic pathway. Therefore, we focused on skull base and optical tract invasion (adjacent to the optical tract or invasion to the orbital apex). We collected multi-institutional data on reirradiation cases to analyze a larger number of cases [11]. Therefore, this study aimed to examine the efficacy and toxicity of reirradiation as a treatment option for the nasal cavity or paranasal sinus tumors.

## 2. Materials and Methods

### 2.1. Patients

Patients (*n* = 78) with recurrent nasal cavity or paranasal sinus tumors treated at five Japanese institutes (Department of Radiology, Graduate School of Medical Science, Kyoto Prefectural University of Medicine; CyberKnife Center, Soseikai General Hospital; Department of Radiation Oncology, Miyakojima IGRT Clinic; Department of Radiation Oncology, Osaka International Cancer Institute; Radiotherapy Department, Fujimoto Hayasuzu Hospital) between 2002 and 2018 were recruited. Patient characteristics are shown in Table 1.

Patients (median age, 65 years; range, 14–87 years) with histologically confirmed recurrent nasal cavity or paranasal sinus cancer after one course of previous radiotherapy with significant overlap were included.

The inclusion criteria were (a) reirradiation as radiotherapy performed after previous radiotherapy of 30 Gy/10 fractions (equivalent 2-Gy fractions = EQD2 ≥ 36 Gy, using α/β = 10) or more, (b) histology confirmed by pathology, (c) pathological or radiological diagnoses obtained for patients presenting for salvage therapy after curative treatment including radiotherapy (definitive or postoperative), (d) no distant metastasis, (e) an Eastern Cooperative Oncology Group performance status of 0 to 2, and (f) recurrence after curative treatment including surgery or chemotherapy with radiotherapy. Patients with (a) metastasis or (b) lymph node metastasis without local failure, and (c) palliative radiotherapy for symptomatic relief (e.g., 6–8 Gy/1 fraction) were excluded.

The data analyzed include demographics (age and sex); tumor characteristics (primary or primary plus lymph node metastasis; site; size and volume; skull base invasion; location adjacent to the optic pathway; treatment (surgical history, chemotherapy, previous radiotherapy, duration from the previous radiotherapy to reirradiation, reirradiation dose, fraction, and treatment duration); and outcome (tumor control and lymph node metastasis, distant metastasis, and survival). We divided the patients into two groups according to the reirradiation dose (prescribed dose in EQD2 ≥ 40 Gyor < 40 Gy), respectively. Sixty-eight patients underwent stereotactic radiotherapy (mainly CyberKnife), eight underwent intensity-modulated radiotherapy (IMRT), and two underwent 3D-conformal radiation therapy (CRT). We did not examine differences in treatment due to low numbers of participants undergoing 3D-CRT or IMRT. Gross tumor volume (GTV) was defined as a visible tumor in imaging studies. An adequate additional margin was added to the GTV to form the planning target volume (PTV). Patients were treated with a median dose of 30 Gy (range, 18–66.7 Gy) in a median of eight fractions (range, 3–30 Gy). EQD2 of previous, present, and accumulated radiotherapy was estimated according to the following equation: EQD2 = n × d × ([α/β] + d)/([α/β] + 2), where n is the number of treatment fractions, d is the dose per fraction in Gy, and α/β = 10.

Toxicity was evaluated according to the Common Terminology Criteria for Adverse Events Version 4.0 (https://ctep.cancer.gov/protocolDevelopment/electronic_applications/ctc.htm#ctc_40, last accessed date: 1 September 2021).

### 2.2. Statistical Analysis

All statistical analyses were performed using R-stat [12] and Stat-view 5.0, statistical software (SAS Institute, Cary, NC, USA). Frequencies were analyzed using a chi-square test. Means were compared using Student’s *t*-test for normally distributed data and the Mann–Whitney U-test for skewed data. Survival data and cumulative incidences were estimated using the Kaplan–Meier method and examined for significance using a log-rank test. Cox’s proportional hazard model was used for the multivariate analysis for local control and survival, and a logistic regression model was used for the multivariate analysis of toxicity. Cutoff values were set at the average or median value of each variable unless otherwise stated. Statistical significance was set at *p* < 0.05.

## 3. Results

### 3.1. Local Control Rate and Overall Survival Rate

**Figure 1:**
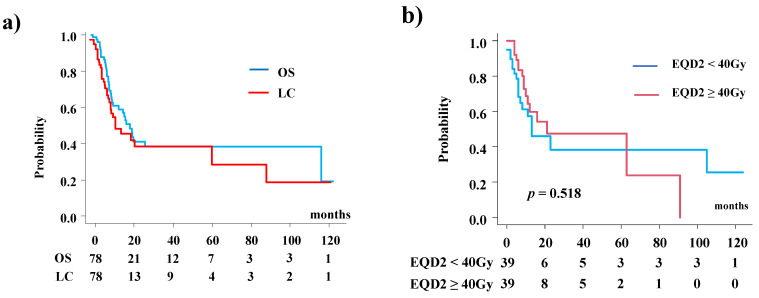

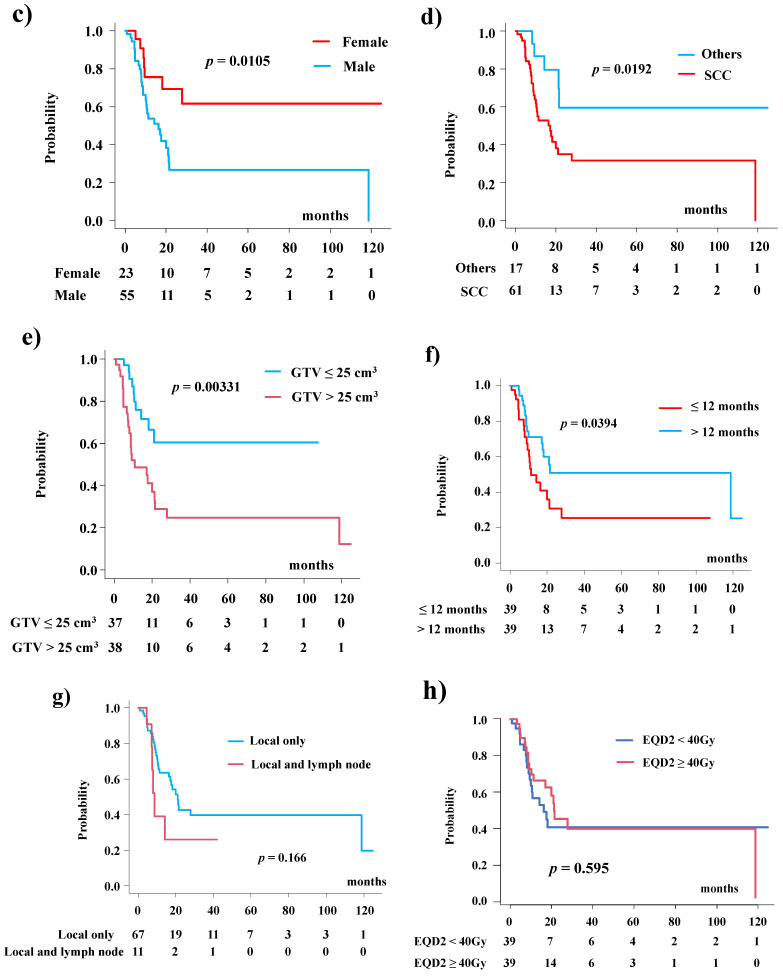

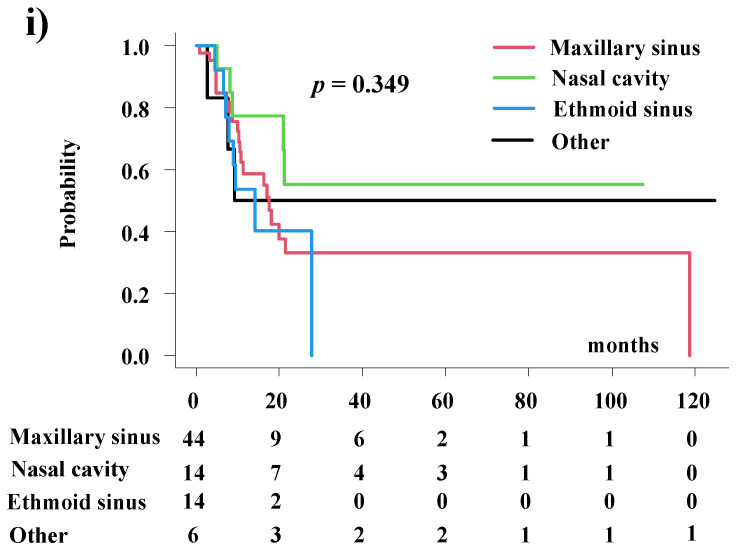
Local control and overall survival rate after reirradiation for nasal cavity or paranasal sinus tumor. (**a**) Total population. Local control (LC) and survival rate (OS). (**b**) Local control rate according to prescribed dose. (**c**) Overall survival according to gender. (**d**) Overall survival according to histology. (**e**) Overall survival according to gross tumor volume (GTV). (**f**) Overall survival according to interval between previous radiotherapy and reirradiation. (**g**) Overall survival according to lymph node involvement. (**h**) Overall survival according to prescribed dose. (**i**) Overall survival according to primary site.

With a median follow-up period of 10.7 months, the median survival time was 20 months (95% confidence interval [CI]: 10.7–118.7 months). The 1- and 2-year survival rates were 60.8% (95% CI: 47.8–71.5%) and 41.0% (95% CI: 27.6–53.9%), respectively (Figure 1a). The 1- and 2-year local control rates were 55.0% (95% CI: 41.2–66.7%) and 39.0% (95% CI: 24.6–53.0%), respectively (Figure 1a). The 2-year local control rates were 38.3% (18.75–57.6%) and 47.5% (25.78–66.4%) between patients treated with prescribed dose EQD2 < 40 Gy and EQD2 ≥ 40 Gy (*p* = 0.518, Figure 1b).

As shown in Table 2, male sex (hazard ratio (HR) 2.99, 95% CI: 1.15–7.75, *p* = 0.024; Figure 1b), GTV (>25 cm^3^) (HR 4.35, 95% CI: 1.79–10.61, *p* = 0.0012; Figure 1c), histology (SCC; HR 4.18, 95% CI: 13.4–13.05, *p* = 0.014; Figure 1d), interval between treatments (≤12 months; HR 2.5, 95% CI: 1.041–6.25, *p* = 0.04; Figure 1e), and lymph node involvement (HR 3.36, 95% CI: 1.20–9.40, *p* = 00021; Figure 1f) were associated with decreased survival in multivariate analysis. No significant correlation was found between overall survival (OS) and tumor location (maxillary sinus or not), prescribed dose (EQD2 < 40 Gy, EQD2 ≥ 40 Gy), lymph node involvement, location adjacent to the optic nerve, or skull base invasion.

Females showed a superior 2-year survival rate (75.7%, 95% CI: 51.0–89.1%) compared with males (53.8%, 95% CI: 37.1–67.1%, *p* = 0.0105; Figure 1b). Patients with SCC histology showed a 2-year survival rate of 35.0% (95% CI: 20.7–49.6%), which was lower than that of patients with other histologies (59.6%, 95% CI: 27.2–81.4%, *p* = 0.0192). Patients with GTV > 25 cm^3^ showed a lower 2-year survival rate (28.8%, 95% CI: 10.6–41.8%) than those with GTV ≤ 25 cm^3^ (60.4%, 95% CI: 37.6–77.1%, *p* = 0.00331; Figure 1c). A longer interval period between previous radiotherapy and reirradiation showed a superior 2-year survival rate (50.8%, 95% CI: 31.1–67.5%) than a shorter interval (30.7%, 95% CI: 14.0–49.2%, *p* = 0.0394). The 2-years OS were 39.2% (21.3–56.7%) and 44.0% (25.0–61.4%) between patients treated with prescribed dose EQD2 < 40 Gy and EQD2 ≥ 40 Gy (*p* = 0.595, Figure 1h, Table 2).

The 2-year survival rate for various tumors was as follows: 33.1% (95% CI: 16.1–51.2%) for maxillary sinus tumors, 55.3% (95% CI: 22.2–79.2%) for nasal cavity tumors, 40.4% (95% CI: 12.4–67.5%) for sphenoid sinus tumors, and 50.0% (95% CI: 11.1–80.4%) for other tumors (Figure 1g, *p* = 0.349).

**Figure 2:**
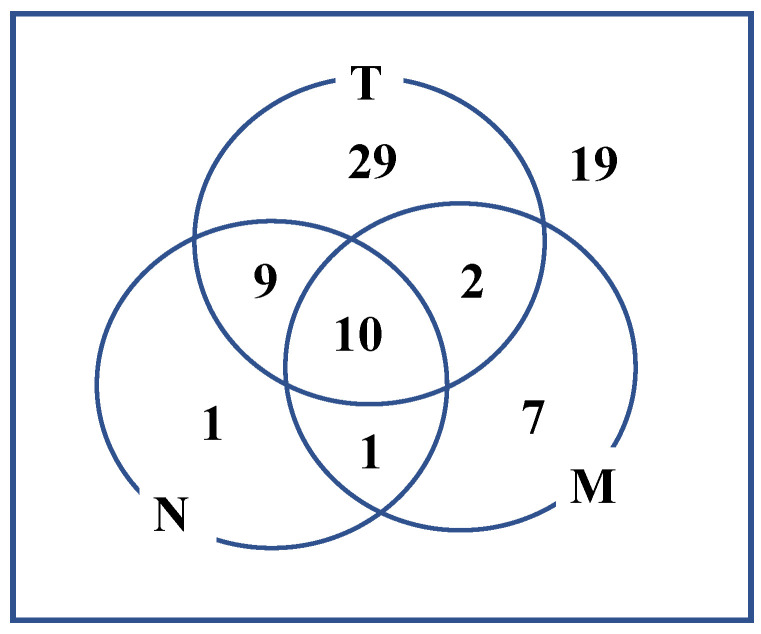
Failure pattern of nasal cavity or paranasal sinus tumor after reirradiation.

The failure patterns are shown in Figure 2. We found 20 metastases in 78 patients (25.6%). We observed a higher incidence of metastasis in lymph node-positive cases (5/11 (45.5%)) than in lymph node-negative cases (15/67 (22.4%)), although the difference was not significant owing to the small number of cases (*p* = 0.210). Nodal involvement without local failure was rare (2.5%), and distant metastasis without local or lymph node metastasis was observed in seven cases (8.9%). There were eight cases with distant metastasis in the lung (including hilar lymph nodes), five in the bones, two in the liver (including abdominal lymph nodes), three in the brain, one in the skin, and one in the stomach, including multiple site involvement.

### 3.2. SCC Subgroup Analysis

**Figure 3:**
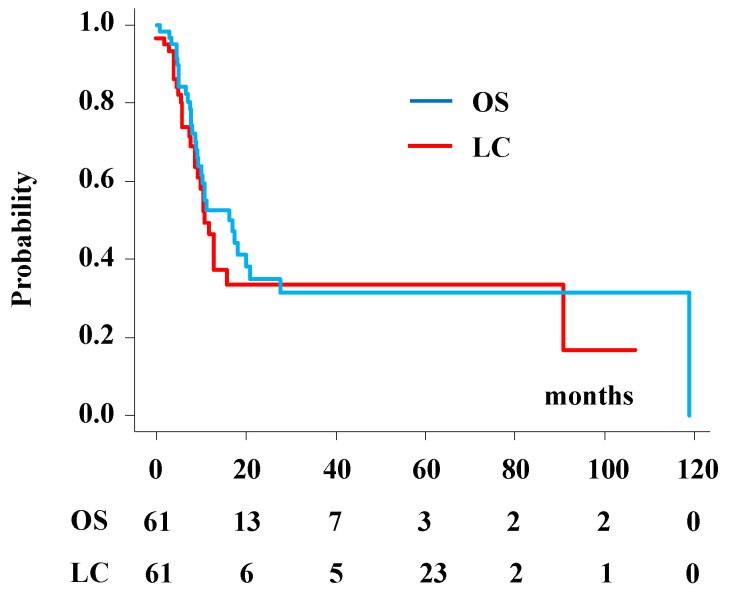
Local control and overall survival rate after reirradiation for squamous cell carcinoma of the nasal cavity or paranasal sinus.

The 1- and 2-year survival rates were 52.8% (95% CI: 37.8–65.5%) and 35.0% (95% CI: 20.7–49.6%), respectively, with a median survival time of 16.3 months (95% CI: 9.4–21 months) (Figure 3). The 1- and 2-year local control rates were 46.4% (95% CI: 30.5–60.8%) and 33.4% (95% CI: 18.6–8.9%), respectively (Figure 3). Sex (HR 3.19, 95% CI: 1.19–8.57, *p* = 0.021, female favorable) and GTV (HR 3.66, 95% CI: 1.55–8.66, *p* = 0.0032, GTV ≤ 25 cm^3^ favorable) were predisposing factors for survival (Table S1). No significant correlation was found between OS and age, previous surgery, chemotherapy, the interval between treatment (first radiotherapy and reirradiation), prescribed dose, skull base invasion, or location adjacent to the optic pathways.

### 3.3. Toxicity

Nineteen patients experienced grade ≥ 3 (22%) toxicity, including five with grade 4 (8.6%) and one with grade 5 symptoms (1.2%) (Table 3). Location adjacent to the optic nerve was the only significant predisposing factor for grade ≥3 toxicity, with an odds ratio of 8.69 (95% CI: 2.19–34.40, *p* = 0.0021) (Table S3). No significant correlation was found between grade ≥ 3 toxicity and age, sex, histology, previous surgery, chemotherapy, size of PTV, interval between treatment (first radiotherapy and reirradiation), prescribed dose (EQD2 < 40 Gy, or EQD2 ≥ 40 Gy), skull base invasion, or location adjacent to the optic pathways. Visual disorder, a grade ≥ 3 toxicity, occurred only in patients with tumors adjacent to the optic pathway without tumor control. Detailed patient characteristics and comparison of toxicity between EQD2 < 40 Gy and EQD2 ≥ 40 Gy group is shown in Tables S3–S5. Therefore, a tumor located adjacent to the optic pathway can be considered a significant predisposing risk factor for grade ≥3 toxicity (Table S2). Grade ≥ 3 toxicity without tumor progression was observed in five patients (6.4%), including grade 5 trismus and grade 3 fistula (skin), nasal bleeding and ulceration, and bone necrosis.

## 4. Discussion

We analyzed the efficacy and toxicity of reirradiation of the nasal cavity or paranasal sinus tumors. In most reports, reirradiation involves all head and neck lesions, not specifically the nasal and paranasal sinuses [6,7,8]. Therefore, to the best of our knowledge, this is the largest sample size study of localized reirradiation of the nasal cavity or paranasal sinus. Our data indicated that reirradiation of nasal and paranasal sinus tumors is feasible and effective. Advancement of radiotherapy technology during the past decade has resulted in a growing number of cases of reirradiation, as evidenced by more patients receiving reirradiation with state-of-the-art equipment, such as stereotactic radiotherapy, IMRT, particle therapy, and brachytherapy [11]. The most common reirradiation sites were the brain (17%), chest (18%), abdomen (16%), head and neck (16%), and vertebral body (11%) in 2018 [11].

Advances in external radiotherapy with IMRT and image-guided radiation therapy have significantly reduced toxicity and improved outcomes. In general, reirradiation (with or without chemotherapy) has been successfully performed without causing excessive morbidity [11,13,14]. Head and neck cancers often have grim prognoses; therefore, we sometimes use a palliative approach to avoid toxicity, hindering active management [11]. Adequate risk assessment before reirradiation of potential complications in normal tissue is difficult because there is insufficient data on recovery from radiation injury [15]. Acute reactions improve within a few months, and retreatment or irradiation has been shown to be tolerable [15,16]. However, late-responding tissues have shown differing radiation recovery patterns on an organ-by-organ basis. The heart, kidney, and bladder do not demonstrate long-term recovery, whereas organs such as the skin, mucosa, lungs, and spinal cord demonstrate long-term recovery [16]. The possibility of unacceptably high severe toxicity, even leading to lethal catastrophic arterial rupture or carotid blow-out syndrome [6], should be anticipated. Recently, a few dose-volume constraints for each organ at risk from reirradiation have been proposed [16,17], one of which included considering temporally-based dose recovery parameters or “discount” factors with time [16]. When considering reirradiation, radiation oncologists must make difficult clinical decisions depending mainly on their own experiences due to the lack of high-level evidence for reirradiation. There are several considerations to help in decision-making, such as (i) normal tissue tolerability [11,16,17], (ii) availability of technical data from the first radiotherapy, (iii) radical or palliative intent related to the prognosis, and (iv) reirradiation schedule including the dose fractionation-volume relationship. Such decisions must be made by multidisciplinary teams with expertise in radiotherapy and implemented on carefully selected patients. Paradis et al. proposed using a consultation team of medical physicians in reirradiation cases [16]. In our department, we used reirradiation only for one patient (1/5 = 20%) with local recurrence at nasal cavity or paranasal sinus without metastasis during 2017–2021.

Most cases of recurrence of nasal cavity or paranasal sinus tumors are highly advanced at the time of diagnosis, are accompanied by symptoms such as visual defects and cranial nerve deficits or a visible mass at the T3–T4 levels, and have poor outcomes. The presence of large air spaces and the fast-growing nature of these tumors influence their growth in multiple sinuses in most cases. Due to their close anatomic relationship with the optic pathway and brain, it is challenging to manage advanced nasal and paranasal sinus tumors.

In newly diagnosed cases, conventional radiotherapy for treating paranasal sinus tumors results in high unilateral or bilateral blindness rates (60% and 10% of patients, respectively) [18,19]. However, advanced radiotherapy, IMRT, image-guided radiotherapy, and stereotactic radiotherapy (SBRT) allow the application of high doses to complex volumes while sparing the surrounding organs at risk, with low toxicity [13,14]. Several authors have reported their reirradiation experiences with IMRT or SBRT in <10 patients [14,20,21]. Iwata et al. [9] reported the treatment results of 51 patients with locally recurrent nasal and paranasal carcinoma using CyberKnife with a prescribed median dose of 35 Gy (range, 20–41.5 Gy) in 3–5 fractions. They reported a 1-year survival rate of 67% and a grade ≥3 toxicity rate of 23%, which is consistent with our findings (1-year survival rate, 60.4%; grade ≥3 toxicity rate, 23.4%).

We found that non-SCC histology, female sex, smaller GTV, longer duration from the previous radiotherapy to reirradiation, and non-involvement of lymph nodes were favorable predisposing factors for survival. These results corroborate those of a previous study [22] involving all head and neck cancers. Distant metastasis was not rare after reirradiation (25.6%), especially in cases of lymph node involvement, consistent with the findings of a previous study [23] that involved all head and neck cancers.

Toxicity after reirradiation of nasal and paranasal sinus tumors is concerning due to the tumor’s anatomical location adjacent to the optical pathway and skull base. Therefore, limiting the late toxicity of the optic pathway or skull base is crucial to ensure long-term survival. We found that the rate of radiotherapy-related late grade ≥3 toxicity without tumor progression was only 6.4%, whereas that of all grade ≥3 toxicities was 22%, indicating that almost all toxicities were due to tumor progression, which requires treatments that are more effective.

The combination of systemic therapy is important for improving patient outcomes. Recently, the potential for immunotherapy has been emphasized in complicated clinical cases. In 2019, the US Food and Drug Administration approved PD-1 inhibition using pembrolizumab as a first-line treatment for patients with metastatic or unresectable recurrent head and neck SCC [24]. The CheckMate 141 study also revealed its potential for immunotherapy. The median OS with a 2-year follow-up was 7.7 months in patients who received nivolumab and 5.1 months in those who received chemotherapy (HR = 0.68, 95% CI: 0.54–0.86). Overall, the 2-year CheckMate 141 data demonstrated that nivolumab not only improved OS at the primary analysis but also continued to demonstrate progressively greater benefit than the investigator’s choice with a minimum of 2 years of follow-up, regardless of PD-L1 expression level [24]. Thus, reirradiation could be more effective in combination with immunotherapies, as radiotherapy could enhance the efficacy of immunotherapy by modulating the immune system [25].

In cases of nasal cavity or paranasal sinus tumors with no previous treatment history, charged-particle therapy has gained attention for its superior dose distribution over that of photon therapy. Patel et al. conducted a systematic review and meta-analysis and concluded that charged-particle therapy could be associated with better outcomes in patients with malignant diseases of the nasal cavity or paranasal sinus [2]. The American Society for Radiation Oncology reported that skull base tumors have good indications for particle therapy in 2013 and sinus cancer in 2017 [26]. In Japan, charged particle radiotherapy for nasal cavity or paranasal sinus tumors has been covered by national insurance since January 2018 owing to its favorable outcomes [27,28]. Several authors have reported good outcomes of reirradiation using particle therapy, with a 2-year survival rate of 60–68% [29,30] (Table 4). Games et al. conducted a systemic review on reirradiation with charged particle beam therapy for treating recurrent skull base and head and neck tumors [31]. They concluded that curative intent skull base and head and neck reirradiation with charged particle radiotherapy is feasible and safe in well-selected cases and is associated with comparable or potentially improved local control and toxicity rates than those obtained using photon radiotherapy. They summarized that the 2-year OS rates of different treatments were: 33–80%, proton: 59–82%, carbon: 14–58%, SBRT: 12–68%, IMRT: 11–81%, 3D-CRT. Grade 3 toxicity was 0–33% for proton, 0–37% for carbon, 0–18% for SBRT, 15–48% for IMRT, and 21–59% for 3D-CRT, although the study was not limited to nasal cavity or paranasal sinus tumors. However, indications for particle beam therapy are limited to selected curative cases with favorable prognoses, and palliative use is beyond the scope of this modality [29,30,31]. In addition, there are several barriers to using particle therapy, such as high cost and low accessibility.

This study has some limitations. First, its retrospective nature, limited follow-up time, and small sample size may limit its application. Second, variables other than the prescribed dose, meticulous dosimetric factors for tumor control and organs at risk (V46 Gy, etc.) [32,33], and non-dosimetric factors (performance status, underlying comorbidity, large vessel invasion, preexisting symptoms, surgery (transurethral resection of the prostate), and fractionation, including daily versus every other day irradiation) were not considered [23,33]. Third, retrospective databases may not record toxicity and tumor control outcomes and may thus have ambiguous data owing to the heterogeneous follow-up periods. Finally, our data included treatment with both curative intent and palliative reirradiation, which made the comparison with other curative treatments difficult.

## 5. Conclusions

Reirradiation of nasal and paranasal sinus tumors is feasible and effective. However, adverse events, including disease-related toxicities, were significant. In addition, a relatively high incidence of distant metastasis was observed after reirradiation, especially in patients with lymph node metastasis. Prognostic factors are emerging to guide multidisciplinary approaches and clinical trial designs.

## Figures and Tables

**Table 1:** Patient characteristics.

Variables	Group	(*n* = 78)
Age		65.00 [14.00, 87.00]
Sex	Female	23 (29.5)
	Male	55 (70.5)
Primary site	Maxillary sinus	44 (56.4)
	Frontal sinus	3 (3.8)
	Sphenoid sinus	3 (3.8)
	Nasal cavity	9 (11.5)
	Nasal cavity + other	5 (6.4)
	Ethmoid sinus	14 (17.9)
Disease site	Primary only	67 (85.9)
	Primary + Lymph node	11 (14.1)
Histology	Squamous cell carcinoma	61 (78.2)
	Chordoma	3 (3.7)
	Melanoma	3 (3.7)
	Olfactory neuroblastoma	2 (2.5)
	Sarcoma	2 (2.5)
	Other	7 (8.9)
Chemotherapy	Yes	27 (34.6)
	No	51 (65.4)
Previous Surgery	Yes	40 (51.3)
	No	38 (48.7)
Adjacent to Optic pathway	Yes	26 (33.3)
	No	52 (66.7)
Skull base invasion	Yes	36 (46.2)
	No	42 (53.8)
Gross tumor volume (GTV)	(cm^3^)	26.40 [1.40, 201.00]
Planning target volume (PTV)	(cm^3^)	33.1 [2.20, 228.70]
Prescribed dose	(Gy)	30.00 [18.00, 66.70]
fractionation	(fractions)	8.00 [3.00, 30.00]
EQD2	(Gy)	38.67 [20.40, 74.70]
Interval between treatment	(months)	12.15 [1.30, 359.80]
Previous prescribed dose	(Gy)	60.00 [27.00, 116.00]
Previous fractionation	(fractions)	30.00 [5.00, 60.00]
Previous EQD2	(Gy)	60.00 [36, 120.00]

Data are presented as patients’ number (%) or median [range] values.

**Table 2:** Multi-variate analysis for survival rate using Cox proportional hazards model.

Variable	Strata	Multivariate Analysis		2-Years OS (95% CI)	*p*-Value
		Hazard Ratio (95% CI)	*p*-Value		
Age, years	(Sequential)	1.01 (0.98–1.05)	0.57		
Sex	Female	1 (referent)	-	75.7% (51.0–89.1%)	
	Male	2.99 (1.15–7.75)	**0.024**	53.8% (37.1–67.1%)	**0.0105**
Location	Maxillary sinus	1 (referent)	-		
	Other	0.70 (0.30–1.64)	0.41		
Histology	Other	1 (referent)	-	59.6% (27.2–81.4%)	
	scc	4.18 (1.34–13.05)	**0.014**	35.0% (20.7–49.6%)	**0.0192**
Lymph node involvement	No	1 (referent)	-	42.7% (27.9–56.7%)	
	Yes	3.36 (1.20–9.40)	**0.021**	26.0% (38.8–57.2%)	0.166
Previous Surgery	No	1 (referent)	-		
	Yes	0.48 (0.20–1.16)	0.1		
Chemotherapy	No	1 (referent)	-		
	Yes	0.56 (0.23–1.40)	0.22		
Goss tumor volume (GTV)	≤25 cm^3^	1 (referent)	-	60.4% (37.6–77.1%)	
	>25 cm^3^	4.35 (1.79–10.61)	**0.0012**	28.8% (10.6–41.8%)	**0.00331**
Interval between treatment	≤12 months	1 (referent)		50.8% (31.1–67.5%)	
	>12 months	2.5 (1.041–6.25)	**0.04**	30.7% (14.0–49.2%)	**0.0394**
Prescribed dose	EQD2 ≥ 40 Gy	1 (referent)	-	39.2% (21.3–56.7%)	
	EQD2 < 40 Gy	1.02 (0.53–1.97)	0.95	44.0% (25.0–61.4%)	0.595
Adjacent to Optic pathway (%)	No	1 (referent)	-		
	Yes	1.11 (0.47–2.63)	0.81		
Skull base invasion	No	1 (referent)	-		
	Yes	0.99 (0.45–2.18)	0.98		

Bold values indicate statistical significance. Abbreviations; CI = confidence interval, OS = overall survival, scc = squamous cell carcinoma.

**Table 3:** Early and late toxicity.

**Frequency of Toxicity**		
**Toxicity Grade**	**Number of Patients**	**(%)**
0	26	(33.3%)
1	2	(2.6%)
2	33	(42.3%)
3	11	(14.1%)
4	5	(6.4%)
5	1	(1.3%)
**Detail of Toxicity Grade ≥3 after Reirradiation**		
**Toxicity**	**Number of Patients**	**(%)**
Meningitis	2	(2.6%)
Mucositis	2	(2.6%)
Visual disorder/blindness	6 (5 Grade 4)	(7.7%)
Bone necrosis	2	(2.6%)
Soft tissue necrosis	1	(1.3%)
Trismus	1 (Grade 5)	(1.3%)
Hemorrhaging	5	(6.4%)
Fistula	5	(6.4%)
Abscess	1	(1.3%)

Some patients had two or more toxicities.

**Table 4:** Summary of studies using particle beam radiotherapy for reirradiation of nasal cavity or paranasal sinus.

Author, Publish Years	Prescription Dose	No. PT	Median Follow-Up (Months)	2-Years Local Control Rate/2-Years OS	Toxicity Grade ≥ 3
Hu, W. [29] 2020	Proton and Carbon 63 Gy [RBE] /21fr, 71 Gy [RBE]/33fr etc.	30 (111 ^1^)	20.2 ^1^	Not available 77% ^2^	3.6% (1 grade 4) ^1^
Hayashi, K. [30] 2019	Carbon 57.6 Gy [RBE] 54.0 Gy [RBE]	31 (48 ^3^)	27.1 ^3^	40.5% ^3^ 59.6% ^3^	10.4% acute toxicities ^3^ 37.5% late toxicities (1 Grade 5)
Current study	Photon	78	10.7	43% 44%	22% (5 grade 4, 1 grade 5)

^1^ including all 111 patients with 81 RT-naive case, ^2^ obtained from figure, ^3^ including entire 48 patients with other sites of tumor.

## Data Availability

The data can be obtained from the author upon reasonable request.

## Supplementary Materials

The following are available online at https://www.mdpi.com/article/10.3390/cancers13246315/s1, Table S1: Multi-variate analysis for survival rate in squamous cell carcinoma using Cox proportional hazards model, Table S2: Multi-variate analysis for toxicity Grade ≥3 using logistic regression model, Table S3: Patient characteristics according to prescribed dose, Table S4: Correlation between prescribed dose and toxicity, Table S5: Correlation between prescribed dose and toxicity profiles.
