# Supplementary material for: Reirradiation for Nasal Cavity or Paranasal Sinus Tumor—A Multi-Institutional Study
Source: Cancers (Basel). 2021 Dec 16;13(24):6315. doi: 10.3390/cancers13246315 (PMC8699758; doi:10.3390/cancers13246315)
Supplement: Supplementary file 1 [file cancers-13-06315-s001.zip › cancers-1433747-supplementary.pdf]

cancers

MDPI

Supplementary Materials

Reirradiation for Nasal Cavity or Paranasal Sinus Tumor—A Multi-Institutional Study

Hideya Yamazaki Gen Suzuki, Norihiro Aibe, Makoto Yasuda, Hiroya Shiomi, Ryoong-Jin Oh, Ken Yoshida, Satoaki Nakamura, Koji Konishi and Mikio Ogita

Table S1. Multi-variate analysis for survival rate in squamous cell carcinoma using Cox proportional hazards model.

Variable	Strata	Multivariate Analysis	
		**Hazard Ratio (95% CI)**	***p* Value**
Age, years	(Sequential)	1.00 (0.96–1.03)	0.94
Gender	Female	1 (referent)	-
	Male	3.19 (1.19–8.57)	**0.021**
Surgery	No	1 (referent)	-
	Yes	0.44 (0.18–1.11)	0.082
Chemotherapy	No	1 (referent)	-
	Yes	0.52 (0.19–1.41)	0.2
Goss tumor volume (GTV)	≤ 25 cc	1 (referent)	-
	25 cc <	3.66 (1.55–8.66)	**0.0032**
Interval between RT	≤ 12 months	1 (referent)	-
	12 months <	0.46 (0.19–1.13)	0.089
Prescribed dose	EQD2 ≤ 40 Gy	1 (referent)	-
	EQD2 > 40 Gy	0.95 (0.46–1.94)	0.89
Adjacent to Optic pathway	No	1 (referent)	-
	Yes	1.55 (0.61–3.91)	0.36
Skull base invasion	No	1 (referent)	-
	Yes	0.76 (0.31–1.86)	0.54

Bold values indicate statistically significance. Abbreviations; CI = confidence interval.

Table S2. Multi-variate analysis for toxicity Grade ≥ 3 using logistic regression model.

Variable	Strata	Multivariate Analysis	
		**Odds Ratio (95% CI)**	***p* Value**
Age, years	(Sequential)	0.98 (0.93–1.03)	0.36
Gender	Female	1 (referent)	-
	Male	1.47 (0.35–6.19)	0.6
Histology	other	1 (referent)	-
	scc	1.52 (0.27–8.51)	0.63
Lymph node involvement	No	1 (referent)	-
	Yes	1.92 (0.38–9.72)	0.43
Surgery	No	1 (referent)	-
	Yes	1.35 (0.35–5.27)	0.66
Chemotherapy	No	1 (referent)	-
	Yes	1.66 (0.42–6.60)	0.47
Planning target volume (PTV)	≤ 25 cm^3	1 (referent)	-
	25 cm^3 <	1.53 (0.39–5.99)	0.54
Interval between RT	≤ 12 months	1 (referent)	-
	12 months <	1.96 (0.47–8.16)	0.36
Prescribed dose	EQD2 ≤ 40Gy	1 (referent)	-
	EQD2 > 40Gy	1.20 (0.46–3.17)	0.71
Adjacent to Optic pathway	No	1 (referent)	-
	Yes	8.69 (2.19–34.40)	**0.0021**
Skull base invasion	No	1 (referent)	-
	Yes	1.32 (0.36–4.83)	0.67

Bold values indicate statistically significance. Abbreviations; CI = confidence interval.

Table S3. Patient characteristics according to prescribed dose.

Variable	Strata	EQD2Gy <40Gy	EQD2Gy ≥40Gy	p - value
		(*n* = 39)	(*n* = 39)	
Age, years	(Sequential)	66.00 [40.00, 87.00]	63.00 [14.00, 84.00]	0.24
Gender	Female	11 (28.2)	12 (30.8)	1
	Male	28 (71.8)	27 (69.2)	
Histology	other	31 (79.5)	30 (76.9)	1
	scc	8 (20.5)	9 (23.1)	
Lymph node involvement	No	32 (82.1)	36 (92.3)	0.310
	Yes	7 (17.9)	3 (7.7)	
Surgery	No	25 (64.1)	13 (33.3)	**0.012**
	Yes	14 (35.9)	26 (66.7)	
Chemotherapy	No	26 (66.7)	25 (64.1)	1
	Yes	13 (33.3)	14 (35.9)	
Previous prescribed dose		60 [30.1, 116.8]	60 [37.5, 120]	0.3874
Interval between RT	months	7.10 [1.30, 157.90]	14.30 [1.40, 359.80]	0.076
Adjacent to Optic pathway	No	26 (66.7)	31 (79.5)	0.307
	Yes	13 (33.3)	8 (20.5)	
Skull base invasion	No	24 (61.5)	18 (46.2)	0.256
	Yes	15 (38.5)	21 (53.8)	
GTV	cc	23.85 [2.20, 201.00]	27.0 [1.4–156.5]	0.9198

Table S4. Correlation between prescribed dose and toxicity.

Toxicity Grade	EQD2 < 40Gy	(%)	EQD2 ≥ 40Gy	(%)	*p* Value
	(*n* = 39)		(*n* = 39)		
0	13	(33.3%)	13	(33.3%)	0.6501
1	2	(5.1%)	1	(2.6%)	
2	15	(38.5%)	16	(41.0%)	
3	7	(17.9%)	4	(10.3%)	
4	2	(5.1%)	5	(12.8%)	

Table S5. Correlation between prescribed dose and toxicity profiles.

Toxicity	EQD2 < 40Gy	EQD2 ≥ 40Gy
	(*n* = 39)	(*n* = 39)
Meningitis	2	0
Mucositis	1	1
Visual disorder/ blindness	2	4
Bone necrosis	0	2
Soft tissue necrosis	0	1
Trismus	0	1
Hemorrhaging	2	3
Fistula	2	3
Abscess	1	0
